# Effects of Life style factors on the symptoms of gastro esophageal reflux disease: A cross sectional study in a Pakistani population

**DOI:** 10.12669/pjms.36.2.1371

**Published:** 2020

**Authors:** Shahid Ahmed, Sajjad Jamil, Hafeezullah Shaikh, Maryam Abbasi

**Affiliations:** 1 Shahid Ahmed, Associate Professor, Department of Medicine, Darul Sehat Hospital, Karachi, Pakistan; 2 Sajjad Jamil, Consultant, Department of Gastroenterology, Liaquat National Hospital & Medical College, Karachi, Pakistan; 3 Hafeezullah Shaikh, Assistant Professor, Department of Gastrenterology, National Institute of Liver and GI Diseases, Dow University of Health Sciences, & Consultant Zubaida Medical Center, Karachi, Pakistan; 4 Maryam Abbasi, Research Coordinator, Darul Sehat Hospital, Karachi, Pakistan

**Keywords:** Lifestyle, Gastroesophageal reflux, Smoking, Sleep deprivation, Exercise

## Abstract

**Background &Objective:**

Gastro esophageal reflux disease (GERD) broadly includes the whole spectrum of reflux disease symptoms like heartburn or acid regurgitation to endoscopic, reflux esophagitis or Barrett’s esophagus. Our aim therefore was to study the association between Gastroesophageal reflux disease symptoms and various lifestyle factors.

**Methods:**

A descriptive cross-sectional study was carried out in the outpatient department of Darul Sehat Hospital, Zubaida Medical Center and Liaquat National Hospital & Medical College, Karachi, Pakistan from January 2018 to October 2018. The selected candidates were asked to fill a validated GERD questionnaire and they were also asked about their lifestyle factors. Odds ratio and their 95% confidence interval were estimated using binary logistic regression with GERD symptoms as the study outcome.

**Results:**

A total of 2000 respondents completed the questionnaire. 69.3% gastroesophageal reflux disease cases were found in participants above 35 years of age while 56.9% subjects were male. The most common lifestyle factors associated with GERD were less exercise time (90.9%) (OR, 6.47; 95% CI, 4.91-8.53), 78.3% participants had habit of eating midnight snacks (OR, 5.08; 95% CI, 4.03-6.40), 87.3% participants reported less interval between dinner and sleep (OR, 6.98; 95% CI, 5.36-9.08). The most important factor relieving GERD symptoms was raising the head of bed during sleep (79.4%) while 43.3% subjects with the habit of post dinner walk reported fewer symptoms of GERD.

**Conclusion:**

Lifestyle factors particularly less physical activity, late evening meals, inadequate sleep, smoking and post dinner lying were found to be associated with GERD symptoms.

## INTRODUCTION

Gastro esophageal reflux disease (GERD) broadly includes the whole spectrum of reflux disease from intermittent symptoms like heartburn or acid regurgitation to endoscopic, reflux esophagitis or Barrett’s esophagus.^1^ About 10-20% of population in western countries report weekly symptoms of GERD.^2^ A study has shown the prevalence of GERD to be 18.1-27.8% in North America, 8.8-25.9% in Europe, 2.5-7.8% in East Asia, 8.7-33.1% in the Middle East, 11.6% in Australia and 23.0% in South America.^3^ In Pakistan it is more common in urban population.^4^ GERD is characterized by reflux of acid, bile, pepsin and pancreatic enzymes due to transient lower esophageal sphincter relaxations and other lower esophageal sphincter pressure abnormalities. This leads to mucosal injury. Other factors contributing to the pathophysiology of GERD include hiatal hernia, impaired esophageal clearance, delayed gastric emptying and impaired mucosal defensive factors.^5^ GERD has a wide range of clinical manifestations from symptoms like chest pain, hoarseness of voice, sore throat, dry cough, burning sensation in chest, food regurgitation to alarming symptoms like dysphagia, odynophagia, anemia and weight loss.

These symptoms can lead to possible complications, including erosive esophagitis, ulcerations, strictures and gastrointestinal bleeding if remain untreated.^6^ According to the American College of Gastroenterology (ACG) the commonly applied diagnostic tools for GERD are upper GI endoscopy, esophageal manometry and ambulatory 24 hour pH monitoring. As suggested by ACG lifestyle measures for GERD are elevation of the head of bed, decrease fat intake, cessation of smoking and avoiding recumbence for three hours post prandial.^7^ Most of the literature showing the association between GERD and lifestyle factors is from the western world. In South Asia limited relevant data are available checking association of GERD with only two or three lifestyle factors. To the best of authors’ knowledge, this study is the first of its kind evaluating in detail the association of all the major lifestyle factors with GERD in Pakistan.

## METHODS

A descriptive cross-sectional study was carried out in outpatient department of Darul Sehat Hospital, Zubaida Medical Center and Liaquat National Hospital & Medical College, Karachi, Pakistan from January 2018 to October 2018. A total of 2000 individuals, aged 18 years or above, were included in the study by means of convenient sampling. Participants with history of comorbid like diabetes mellitus, hypertension, acid peptic disease chronic liver disease or chronic use of NSAIDS were excluded. The calculated sample size was 1750 using 24% GERD prevalence,^4^ 95% confidence interval, 5% precision and 80% power which was inflated by 10-12% to account for non-responders. A pilot feasibility study was conducted by administering the questionnaire to 100 participants. Pilot study was conducted both in Urdu and English languages. After obtaining approval from ethical review committee (IRB-010/LCMD/13, Dated 09 February 2017) a validated questionnaire was administered by the volunteers, under direct supervision of the principal investigator. The questionnaire was administered after obtaining written consent of the participants. It comprised of three sections, demographics, “Ritcher Acid Scale “questionnaire for the screening of GERD^4^ and lifestyle factors. A cutoff point of two or greater was selected to confirm the presence of GERD. The participants were divided into two equal groups (GERD positive and GERD negative) using non-probability purposive sampling. Administered questionnaire was adapted from previously published studies.^7^,^8^ Each participant was classified as underweight, normal, over weight and obese according to the cutoff value of BMI used in a previous study conducted on an Asian population.^9^ Age of the study participants was divided into two groups to observe the association of GERD symptoms with increasing age i.e. ≤ 35 years and > 35 years.

Data were entered and analyzed using statistical package for social sciences (SPSS) version 16.0 Frequencies and percentages were calculated for baseline characteristics like gender, age group, ethnicity and body mass index. Independent sample t-test was used to compare the means of two groups whereas Pearson chi square test was used to determine the association of GERD with baseline parameters and also with life style characteristics. Binary logistic analysis was used to develop a risk assessment model for the study outcome. All p-value less than 0.05 were considered statistically significant.

## RESULTS

Out of a total of 2000 individuals included in the study n=842 were males and n=1158 were females. Of male and female individuals, n= 569 (67.7%) and n=431 (37.3%) had GERD respectively. GERD cases were found to be more common in individuals (77.96%) aged more than 35 years. The highest prevalence of GERD found within Pashto ethnicity (28.9%). All socio-demographic characteristics studied were significantly associated with GERD (p<0.05) (Table-I).

**Table-I:** Association of GERD with Baseline Characteristics of studied Samples (n=2000).

Baseline Characteristics		GERD				p-value

		Negative (n=1000)		Positive (n=1000)		

		N	%	N	%	
Gender	Male (n=842)	272	32.34	569	67.7	<0.01*
	Female (n=1158)	727	62.7	431	37.3	
Age	Mean, SD	30.0	10.47	44.73	13.92	<0.01**
Age group	≤35 years (n=1111)	804	72.4	307	27.6	<0.01*
	>35 Years (n=889)	196	22.04	693	77.96	
Ethnicity	Punjabi	181	18.1	118	11.8	<0.01*
	Sindhi	160	16.0	171	17.1	
	Pashto	181	18.1	289	28.9	
	Baluchi	115	11.5	206	20.6	
	Urdu	363	36.3	216	21.6	
BMI	Mean, SD	24.54	5.20	26.42	5.13	<0.01**
BMI (kg/m2)	Normal (18.5-22.9)	322	32.2	184	18.4	<0.01*
	Underweight (<18.5)	65	6.5	22	2.2	
	Overweight (23-26.9)	320	32.0	431	43.1	
	Obese (≥27)	293	29.3	363	36.3	

* p<0.05 was considered significant using Pearson chi square test

** Mean differences considered significant using Independent Sample t-test.

Forty two percent (42.2%) of the participants reported increased weight in the adulthood, 90.9% exercised less than 30 minutes a day, 78.3% had habit of taking midnight snacks, 77.5% said that they had inadequate sleep, 69.4% skipped the breakfast on frequent basis, 87.3% said that they eat dinner within two hours of going to sleep, 42.6% had habit of smoking, 60.6% were smoking for more than five years, 44.3% reported to smoke more than ten cigarettes per day, 5.1% reported drinking alcohol, 13% had habit of chewing pan, 96.5% drank less than eight cups of tea per day, 79.4% accepted that raising head of bed during sleep relieves heartburn, 21.4% said that they increase duration between eating and sleeping to relieve heartburn, 20.8% did walking after dinner, 69.5% used to lie down or walk whereas 30.5% used to sit or be in recumbent position for post dinner physical activity, 11.2% said that they did fifteen or more minutes exercise daily. All of these lifestyle factors were significantly associated with GERD (p<0.05) (Table-II). Binary logistic regression analysis further showed that males had 3.52 times higher risk of GERD as compared to females while older age and increased BMI also increased the risk of GERD.

**Table-II:** Association of GERD with life style behaviors of studied samples (n=2000).

Life Style Behaviors		GERD Negative (n=1000)		GERD Positive (n=1000)		p-value

		N	%	N	%	
Body weight increased in adulthood	Yes	366	36.6	422	42.2	<0.01*
Exercise time less than 30 minutes	Yes	525	52.5	909	90.9	<0.01*
Habit of midnight snacks	Yes	372	37.2	783	78.3	<0.01*
Feeling of inadequate sleep	Yes	448	44.8	775	77.5	<0.01*
Frequent skipping of breakfast	Yes	441	44.1	694	69.4	<0.01*
Dinner within two hours before going to sleep	Yes	504	50.4	873	87.3	<0.01*
Habit of smoking	Yes	86	8.6	426	42.6	<0.01*
Smoking since how many years	≤5 years	62	72.1	169	39.4	<0.01*
	>5 years	24	27.9	260	60.6	
Number of cigarettes daily	≤10	77	89.6	239	55.7	<0.01*
	>10	9	10.5	190	44.3	
Habit of alcohol drinking	Yes	9	0.9	51	5.1	<0.01*
On how many occasions you consumed alcohol in last 2 weeks	1-4 times	9	0.9	51	5.1	<0.01*
Habit of chewing pan	Yes	140	14	130	13	0.531
Cups of tea daily	Never	279	27.9	35	3.5	<0.01*
	≤8	721	72.1	965	96.5	
Raising head of bed during sleep relieve heartburn	Yes	197	19.7	794	79.4	<0.01*
Increasing duration between eating and sleeping relieve heartburn	Yes	291	29.1	214	21.4	<0.01*
Walking after dinner relieve heartburn	Yes	567	56.7	208	20.8	<0.01*
Post dinner physical activity	Lying or Walking	563	56.3	695	69.5	<0.01*
	Sitting or Recumbent	437	43.7	305	30.5	
How long exercise daily	Never	354	35.4	888	88.8	<0.01*
	15 or more minutes	646	64.6	112	11.2	

* p<0.05 was considered significant using Pearson chi square test.

Results for assessing association of various lifestyle factors with GERD further showed that individuals with increased body weight in adulthood, exercising less than 30 minutes a day, habit of taking midnight snacks, having inadequate sleep, skipping breakfast, eating dinner within two hours of going to sleep and habit of smoking were at high risk of GERD.

Furthermore, the multivariate model adjusted for age, gender and BMI of the participants showed that odds of life style risk factors to be associated with GERD decreased as compared to the un-adjusted model, and body weight increase in adulthood became an insignificant risk in the multivariable model (Table-III).

**Table-III:** Odds Ratio Estimation of GERD with Life Style Characteristics using Binary Logistic Regression.

Life Style Characteristics		Univariate Analysis		Multivariate Analysis	

		Odds Ratio	95% Confidence interval	Odds Ratio	95% Confidence interval
Body weight increased in adulthood	Yes	1.26*	1.05,1.51	1.19	0.96,1.49
Exercise time less than 30 minutes	Yes	9.03*	7.04,11.58	6.47*	4.91,8.53
Habit of midnight snacks	Yes	6.09*	4.99,7.42	5.08*	4.03,6.40
Feeling of inadequate sleep	Yes	4.24*	3.49,5.15	3.22*	2.57,4.03
Frequent skipping of breakfast	Yes	2.85*	2.39,3.54	2.70*	2.17,3.35
Dinner within two hours before going to sleep	Yes	6.76	5.40,8.46	6.98*	5.36,9.08
Habit of smoking	Yes	7.88	6.11,10.17	6.25*	4.40,8.91
Habit of alcohol drinking	Yes	5.91*	2.89,12.08	1.68	0.76,3.68
Raising head of bed during sleep relieve heartburn	Yes	15.71*	12.62,19.54	11.95*	9.31,15.32
Increasing duration between eating and sleeping relieve heartburn	Yes	0.66*	0.54,0.81	0.83	0.65,1.06
Walking after dinner relieve heartburn	No	4.98*	4.09,6.07	3.92*	3.12,4.93
Post dinner physical activity	Lying	3.61*	2.09,6.23	2.24*	1.19,4.20
	Walking	0.36*	0.21,0.62	0.25*	0.13,0.47
	Sitting	0.6	0.35,1.05	0.45*	0.24,0.84
How long exercise daily	15 minutes	0.11*	0.08,0.15	0.14*	0.10,0.20
	30 minutes	0.05*	0.03,0.08	0.04*	0.02,0.07
	>30 minutes	0.02*	0.01,0.03	0.02*	0.01,0.03

* p<0.05 was considered significant.^a^ Model were adjusted for Age, Gender and BMI.

Moreover, in the univariate model people who increased duration of sleep time between eating and sleeping were 0.66 times less likely to have GERD (OR, 0.66; 95% CI, 0.54-0.81). Walking as post dinner activity also decreased the odds of having GERD (OR, 0.36; 95% CI, 0.21-0.62). Increased time of exercise on daily basis also decreased the odds of having GERD i.e. for 15 minutes (OR, 0.11; 95% CI, 0.08-0.15), for 30 minutes (OR, 0.05; 95% CI, 0.03-0.08) and for >30 minutes (OR, 0.02; 95% CI, 0.01-0.03) (Table-III).

The study results further showed that 60% cases of GERD reported for the uncomfortable feeling behind the breast bone, 62.4% felt burning sensation in the back of throat, 9.6% reported for a bitter acid taste in mouth, 95.3% cases experienced problem after meal, 47.5% said antacid only provide temporary relief and 44.8% cases of GERD said they taking prescription medication for heart burn still having symptoms (Table-IV).

**Table-IV:** Ritcher acid scale of both GERD and Control Group.

Scale		GERD			

		Negative		Positive	

		n	%	n	%
An uncomfortable feeling behind the breast bone that seems to be moving upward from the stomach.	No	821	82.1	400	40.0
	Yes	179	17.9	599	60.0
A burning sensation in the back of your throat.	No	851	85.1	376	37.6
	Yes	149	14.9	624	62.4
A bitter acid taste in your mouth.	No	889	88.9	904	90.4
	Yes	111	11.1	96	9.6
Do you experience these problems after meal	No	999	99.9	47	4.7
	Yes	1	.1	953	95.3
Heartburn two times per week	No	1000	100.0	178	17.8
	Yes	0	.0	822	82.2
Antacids only provide temporary relief	No	1000	100.0	525	52.5
	Yes	0	.0	475	47.5
Taking prescription medication for heartburn but still having symptoms	No	1000	100.0	552	55.2
	Yes	0	.0	448	44.8

## DISCUSSION

GERD is closely linked to lifestyle factors that facilitate acid reflux by sphincter relaxation or stimulating acid secretions. Most of the data comparing GERD and lifestyle factors are from the western countries. This study focused on the impact of lifestyle factors on GERD in Pakistan. In our study the target population was patients visiting the outpatient department of a tertiary care hospital of Karachi. The study population belonged to diverse ethnicities from rural and urban areas of Pakistan. A study performed in India reported higher prevalence of GERD in urban population with odds ratio of 1.8^10^ though contrary to our findings where the highest prevalence of GERD was found in Pashto speaking individuals (28.9%) who were mostly from the rural areas of Pakistan.

A higher prevalence of GERD was found with increasing age, 69.5% patients who reported GERD symptoms were above 35 years of age (OR, 9.26), confirming the findings of a study conducted in Albania demonstrating a higher prevalence of GERD among older participants (OR 1.85).^11^ This has been supported by other studies highlighting the likelihood of developing GERD in old age.^12^,^13^ On the other hand few studies failed to show any association of GERD with age^14^,^15^ and the idea of developing GERD with increasing age remains controversial.

Higher BMI was found as a risk factor for GERD in the studies from North America^16^ but a study from Bangladesh found no association between GERD and BMI.^17^ In our study though, a significant difference was found in the prevalence of GERD with increasing BMI with odds ratio of 2.35 and 2.16 in overweight and obese participants respectively.

In our study, prevalence estimates of GERD were higher among men than women with odds ratio of 3.52. This can be comparable to the findings of another study conducted in Pakistan.^4^

Recumbence and lying down after meals are known risk factors of GERD.^18^ The protective effect of normal physical activity in GERD has been highlighted in many studies.^19^ It has been demonstrated in our study that GERD symptoms are more prevalent in physically inactive participants with exercise time of less than 30 minutes (OR, 6.47). The odds ratio of post dinner lying was significant (OR, 2.24) when compared with recumbence in our study.

It is a common belief that some dietary habits would aggravate GERD such as dinner within two hours before going to sleep or taking a midnight snack. In our study the odds ratio of developing GERD in participants taking midnight snacks was 5.08 while for dinner within two hours before going to sleep was 6.98. This association has been previously proved in a study conducted in Korea.^20^

Another study showed that individuals with poor quality sleep had more symptoms of GERD.^21^ The exact mechanism for why a subject sleeps fewer hours remain unknown, but gastric motor function is known to be active during sleep.^22^ Thus inadequate sleep could disturb this motor function and may induce GERD. In the present study, 77.5% individuals with GERD reported inadequate sleep (OR, 3.22).

People who stay empty stomach are prone to develop GERD. This was also shown in our study with 69.4% of GERD positive subjects reporting frequent skipping of breakfast (OR, 2.70). Smoking has often been cited as a risk factor for GERD. Although the findings on this matter are inconsistent, several studies showed direct relationship of GERD with smoking.^23^,^24^ In the current study GERD was found to be more common in individuals who smoked with odds ratio of 6.25. In the current study, 79.4% participants reported improvement in the severity of GERD with elevating the head of the bed during sleep.

### Limitations of the study

The limitations of the study include self-reported diagnosis of GERD based on history and not on endoscopic findings. Furthermore, recall bias might have affected the study results because questionnaire required the participants to recall previous events in the past few weeks. Moreover, it is also acknowledged that the use of convenience sampling technique due to resource constraints may have limited the generalizability of the study findings.

## CONCLUSION

The study results confirm that lifestyle factors have a significant association with GERD symptoms. The most common socio-demographic risk factors identified for developing GERD were older age, higher BMI, male gender and rural population. Moreover, the lifestyle factors that may lead to GERD were lack of exercise, taking midnight snacks, skipping breakfast, smoking and lack of sleep. Further interventional studies are needed to see the impact of lifestyle changes in controlling the symptoms of GERD. Effective measures should be taken to spread awareness about lifestyle modifications.

In this study we used non-invasive means of diagnosing GERD which is primarily used in such cases. We can easily use “Ritcher Acid Scale” for identifying individuals at high risk for developing GERD and then they should be counseled about all these lifestyle measures pointed out in our study for preventing the disease.

### Author Contribution:

**SA, HS:** Conceived, designed and did statistical analysis & editing of manuscript.

**SJ, MA:** Did data collection and manuscript writing.

**SA:** Takes the responsibility and is accountable for all aspects of the work in ensuring that questions related to the accuracy or integrity of any part of the work are appropriately investigated and resolved.
